# Intrathecal injection of human umbilical cord blood-derived mesenchymal stem cells for the treatment of basilar artery dissection: a case report

**DOI:** 10.1186/1752-1947-5-562

**Published:** 2011-12-04

**Authors:** Hoon Han, Sang-Keun Chang, Jennifer J Chang, Soo-Han Hwang, Seung-Hyup Han, Bok-Hwan Chun

**Affiliations:** 1 Seoul Cord Blood Bank, Histostem Ltd., Seoul Life Foundation Building, 518-4, Dunchon-dong, Kang Dong-gu, Seoul 134-060, Korea; 2 Department of Neurosurgery, Konkuk University Hospital, Seoul, Korea

## Abstract

**Introduction:**

Basilar artery dissection is a rare occurrence, and is significantly associated with morbidity and mortality. To the best of our knowledge, we report the first case of basilar artery dissection treated with mesenchymal stem cells.

**Case presentation:**

We present the case of a 17-year-old Korean man who was diagnosed with basilar artery dissection. Infarction of the bilateral pons, midbrain and right superior cerebellum due to his basilar artery dissection was partially recanalized by intrathecal injection of human umbilical cord blood-derived mesenchymal stem cells. No immunosuppressants were given to our patient, and human leukocyte antigen alloantibodies were not detected after cell therapy.

**Conclusions:**

This case indicates that intrathecal injections of mesenchymal stem cells can be used in the treatment of basilar artery dissection.

## Introduction

Little has been reported on the clinical manifestations of basilar artery dissections [1]. It has been suggested that rare cells in human umbilical cord blood (hUCB) could be partially induced to express markers of both neurons and glia [2]. A neural stem cell-like subpopulation could be selected and expanded *in vitro *by hUCB cells, which showed a high commitment (about 30% and 40% of the population) to neuronal and astrocytic fates, respectively [3]. In animal models, administration of CD34^+ ^hUCB cells has been shown to enhance neurogenesis via angiogenesis, resulting in the restoration of ischemic areas [4]. We identified that hUCB contained not only hematopoietic stem cells (HSC) but also mesenchymal stem cells (MSC) which expressed neural makers such as Tuj1, TrkA, glial fibrillary acidic protein (GFAP) and cyclic nucleotide phosphodiesterases (CNPases) facilitating a therapeutic approach for neurodegenerative diseases [5]. Therefore, we attempted to treat our patient with basilar artery dissection by intrathecal injection of hUCB-derived MSC.

## Case presentation

**Figure 1:**
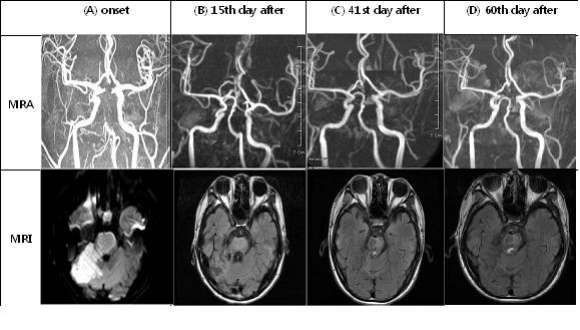
**MRI scans and magnetic resonance angiographic findings before and after first injection of human umbilical cord blood (hUCB)-derived mesenchymal stem cells (MSC)**. **(A) **Partial recanalization of the basilar artery with residual severe stenosis was shown in the subacute infarction sites of bilateral pons, midbrain and right superior cerebellum. **(B) **Bilateral hypertropic olivary degeneration and further recanalization of the basilar artery with residual irregular stenosis was shown in the subacute to chronic infarction sites. **(C) **Luminal irregularity at the mid-to-distal basilar artery with residual stenosis was shown in the chronic infarction sites. **(D) **No intra-cranial lesion was shown in the chronic infarction sites, and effusion size was decreased at the right mastoid.

A 17-year-old Korean man with basilar artery dissection was transferred to our hospital one month after onset of symptoms. Our patient's National Institutes of Health (NIH) stroke scale score was 35(Table 1). His pupils were pinpoint, his light reflex was slightly positive, and movements of his external ocular muscle (EOM) were paralyzed. On cranial nerve examination, our patient showed no response to noxious pain to his motor and sensory nerves. Our patient was in quadriplegia both with a sagging soft palate and a negative gag reflex, and showed a strong clonus of the deep tendon reflex (DTR) (Table 2). Our patient was diagnosed as having a basilar artery dissection based on clinical manifestations and radiological findings, including computed tomography (CT) and MRI scans and magnetic resonance angiography (Figure 1). Because our patient showed no therapeutic response after treatment with antiplatelet drugs and anticoagulants for one month after onset of this disease, an alternative approach with hUCB-derived MSC was applied to our patient. Our patient's human leukocyte antigen (HLA) type was A11,24, B35,61 and DR04,14. Our patient had no history of trauma, hypertension, diabetes mellitus, hyperlipidemia, stroke, seizure, and smoking or drinking. hUCB-derived MSC were prepared according to methods described previously [5]. hUCB-derived mononuclear cells were separated and cultured for a few days. Non-adherent HSC were discarded, and adherent MSC were continued in culture, with two medium changes per week. Samples of 1.2 × 10^7 ^MSC were prepared with each unit of hUCB. Three different units of allogeneic hUCB-derived MSC were intrathecally injected into our patient on the 35th day after onset without using immunosuppressants or antibiotics. Surface markers for MSC injected were CD13^+^, CD29^+^, CD31^+^, CD45^+^, CD73^+^, CD90^+^, CD105^+ ^and CD166^+^; the HLA allele types of the donors were A11,24, B35,62 and DR04,08, respectively. Treatment of patients with hUCB-derived MSC was approved by the Korea Food and Drug Administration (KFDA). The improvement in our patient's clinical symptoms, such as a positive gag reflex and relaxation of muscle tone rigidity, was observed from the fifth day after treatment. The voluntary eyeball movement of our patient was possible on the 27th day after treatment, and the rigidity of his muscle tone was reduced enough for him to sit in a wheelchair (Table 2). The second and third injections of hUCB-derived MSC were performed on the 15th day and 41st day after first treatment, respectively. The HLA allele types of donors on these occasions were A11,24, B27,35 and DR04,14, respectively. No other medical treatment was performed, and the absence of new intracranial lesions was confirmed by MRI scan and magnetic resonance angiography (Figure 1). The improvement of clinical symptoms began to be observed from the fifth day after intrathecal injection of hUCB-derived MSC, and the significant changes from the 15th day after treatment were confirmed by radiological findings. These data were similar to our previous findings that hUCB-derived MSC had high endothelial functions [6].

**Table 1:** Comparison of National Institutes of Health (NIH) stroke scale before and after first injection of human umbilical cord blood (hUCB)-derived mesenchymal stem cells (MSC)

		Onset	41st day after	60th day after
1a	Level of consciousness (LOC)	3	0	0

1b	LOC questions	2	2	2

1c	LOC commands	2	0	0

2	Best gaze	2	1	1

3	Visual	3	1	0

4	Facial palsy	?	2	2

5	Motor arm	Right 4	3+/-	3
		
		Left 4	3+/-	3

6	Motor leg	Right 4	4	3
		
		Left 4	4	3

7	Limb ataxia	Untestable	Untestable	Untestable

8	Sensory	2	1+/-	1

9	Best language	3	2	3

10	Dysarthria	Untestable	Untestable	Untestable

11	Extinction and attention	2	2-	1

Total		35	25	20

**Table 2:** Comparison of neurological findings before and after first injection of human umbilical cord blood (hUCB)-derived mesenchymal stem cells

	Onset	Fifth day after	15th day after	27th day after	41st day after	60th day after
Glasgow coma scale	E1 VT M2	E2 VT M4	E3 VT M4	E4 VT M4	E4 VT M4	E4 VT M4

Extension hypertonic posturing	1+/1+	1+/1+	1+/1+	1+/1+	1+/1+	1+/1+

Ankle clonus	4+/4+	3+/3+	3+/3+	3+/3+	3+/3+	3+/3+

Muscle tone rigidity	4+/3+	4+/3+	4+/3+	2+/2+	2+/2+	2+/2+

Deep tendon reflex	3+/3+	4+/3+	4+/3+	3+/3+	3+/3+	3+/3+

Babinski sign	(-)/(-)	(-)/(-)	(-)/(-)	(-)/(-)	(-)/(-)	(-)/(-)

Nystagmus	(-)	(-)	(-)	(-)	(-)	(-)

External ocular muscle	Gaze paralysis	Gaze paralysis	Gaze paralysis	Gazing 60'	Gazing 60'	Gazing 60'

Pupils	Pinpoint	Pinpoint	Pinpoint	3+/3+	3+/3+	3+/3+

Light response	+/+	+/+	+/+	+/+	+/+	+/+

Facial palsy (central type)	(+)	(+)	(+)	(+)	(+)	(+)

Gag reflex	(-)	(-)	(-)	(+)	(+)	(+)

Soft palate sagging	(-)	(-)	(-)	(+)	(+)	(+)

Anarthria	(+)	(+)	(+)	(+)	(+)	(+)

Dysphagia	(+)	(+)	(+)	(+/-)	(-)	(-)

Quadriplegia	(+)	(+)	(+)	(+)	(+)	(+)

## Discussion

The growth factors and stem cell populations of hUCB could enhance the neurological recovery after stroke by stimulation of neural sprouting and proliferation of endogenous progenitor cells in the brain [7]. It has been proposed that the secretion of various cytokines and growth factors by MSC might play an important role not only in the engraftment of transplanted HSC but also in the neural differentiation of MSC [8]. Our previous *in vitro *study showed that cytokines that were known as novel neurotropic and anti-inflammatory factors such as insulin-like growth factor (IGF)-1, IGF-2, insulin-like growth factor binding protein (IGFBP)-1, IGFBP-2, IGFBP-5, monocyte colony stimulating factor (M-CSF), monocyte chemoattractant protein (MCP)-1, regulated upon activation, normal T cell expressed and presumably secreted (RANTES), macrophage inflammatory protein (MIP)-1α, interferon-inducible protein (IP)-10 and interleukin (IL)-8 were highly detected in the supernatant of cultured hUCB-derived MSC. However, the serum levels of these cytokines before and after treatment were notably not observed in our patient, although it was hypothesized that the functional recovery might be influenced by growth factors (data not shown). It has been reported that brain injuries of animals, caused by stroke and traumatic brain injury (TBI), recovered when hUCB cells were injected into the animal at 24 hours after occurrence of the condition [9]. A few cases in human patients, such as one patient who was treated 19 and half years after spinal cord injury [10] and another patient with Buerger's disease [11], have been cured by transplantation of hUCB-derived MSC. Therefore, it was suggested that the existence of both stem cell-specific cytokines as early effectors and the differentiated tissue-specific cytokines as later effectors could improve clinical symptoms. Because these cytokines might contribute to the rapid neural differentiation of hUCB-derived MSC, their potential usage in the treatment of human neurological disorders, such as basilar artery dissection, were of particular interest. The MRI scan results and magnetic resonance angiographic findings as shown in Figure 1A were observed about one month after onset. Even though partial recanalization of the basilar artery was found before intrathecal injection of hUCB-derived MSC, the magnetic resonance angiographic data of clinical symptoms from the 15th day after first treatment clearly show that recanalization of the basilar artery with residual irregular stenosis had been helped by transplantation of these stem cells.

## Conclusions

In total, nine units of allogeneic hUCB-derived MSC were injected without using immunosuppressants. Each unit had two out of six HLA alleles mismatched to our patient, but the formation of HLA alloantibodies was not detected. It is therefore thought that multiple units of hUCB-derived MSC can be safely applied by intrathecal injection for the treatment of brain infarction.

## Consent

Written informed consent was obtained from the patient's next-of-kin for publication of this case report and any accompanying images. A copy of the written consent is available for review by the Editor-in-Chief of this journal.

## Competing interests

The authors declare that they have no competing interests. HH is the founder of Histostem in Korea, established in 2000. S-HHw, S-HHa and B-HC are staff of Histostem. JJC is a visiting doctor who works at Histostem. S-KC is a professor at the Department of Neurosurgery, Konkuk University Hospital, Seoul, Korea, and has no financial connection with Histostem.

## Authors' contributions

All authors read and approved the final manuscript. HH was involved in drafting the data from our patient, and analyzed the data regarding the disease process. S-HHw, S-HHa and B-HC contributed to the preparation of hUCB-derived MSC. JJC was the attending medical doctor who made a substantial contribution to the analysis of data. S-KC performed the intrathecal injection of hUCB-derived MSC given to our patient.
